# Tensiomyographic Assessment of Contractile Properties in Elite Youth Soccer Players According to Maturity Status

**DOI:** 10.5114/jhk/161571

**Published:** 2023-04-20

**Authors:** Alexis Padrón-Cabo, Francisco J. Corredoira, Miguel Lorenzo-Martínez, Sixto González-Víllora, Ezequiel Rey

**Affiliations:** 1 Department of Physical and Sports Education, Faculty of Sport Sciences and Physical Education, University of A Coruña, A Coruña, Spain.; 2 Faculty of Education and Sport Sciences, University of Vigo, Pontevedra, Spain.; 3 Department of Physical Education, Arts Education, and Music, Faculty of Education, University of Castilla-La Mancha, Albacete, Spain.

**Keywords:** peak height velocity, maturation, stiffness, football

## Abstract

Little is known about how muscle contractile properties are affected by biological maturation in elite youth soccer players. This study aimed to determine the effects of maturation on contractile properties of rectus femoris (RF) and biceps femoris (BF) muscles assessed by tensiomyography (TMG) and provide reference values for elite youth soccer players. One hundred twenty-one elite youth soccer players (14.98 ± 1.83 years; 167.38 ± 10.37 cm; 60.65 ± 11.69 kg) took part in the study. The predicted peak height velocity (PHV) was used in order to establish players' maturity status (Pre-PHV, n = 18; Mid-PHV, n = 37; Post-PHV = 66). Maximal radial displacement of the muscle belly, contraction time, delay time, and contraction velocity for RF and BF muscles were recorded. One-way ANOVA showed no significant differences between PHV groups for any tensiomyography variables in RF and BF muscles (p > 0.05). Our results established that maturity status did not show a significant effect in mechanical and contractile properties on RF and BF muscles evaluated by TMG in elite youth soccer players. These findings and reference values can be useful for strength and conditioning coaches of elite soccer academies in order to optimize the evaluation of neuromuscular profiles.

## Introduction

During talent identification and the selection process of youth soccer players, establishing the key predictors of performance ensure that the most talented players receive high-quality coaching and training conditions (Sarmento et al., 2018). This selection process is extremely complex and depends on the interaction between physical, technical-tactical, and psychological factors (Bergkamp et al., 2019). In the last years, clubs and federations have been interested in complementing subjective assessments with a more scientific approach to increasing the probability of selecting successful players (Bergkamp et al., 2019; Dugdale et al., 2020). Recently, Ford et al. (2020) surveyed a total of 29 professional clubs in relation to the talent identification process. The results of this survey indicated that 72–86% of the respondents claimed to use physiological measurements for talent identification between the ages of 12 and 21 (Ford et al., 2020). Similarly, several systematic reviews have highlighted prognostic relevance to physiological talent predictors for youth soccer players´ future success in adulthood (Murr et al., 2018; Sarmento et al., 2018). Concretely, neuromuscular abilities play an important role in contemporary soccer due to a progressive increase in the number of high-speed actions in professional soccer matches (Barnes et al., 2014). In this regard, Deprez et al. (2015) concluded that most explosive players were more likely to receive a professional contract and even more playing minutes once they reached professional status. Therefore, in order to optimize the identification of talented players, it is necessary to establish their neuromuscular profile.

Soccer players have been universally allocated in chronological age groups, but this situation can cause inequality in talent identification and in multiple aspects of training prescription (Mujika et al., 2009). Scientific literature has determined that individuals with the same chronological age can differ with regard to their biological maturation (Lloyd et al., 2014). In this respect, the influence of biological maturation on physiological performance and neural changes in youth soccer players has been well documented (Brownlee et al., 2018; Murtagh et al., 2018, 2020). Specifically, Murtagh et al. (2018) concluded that acceleration and sprint performance might be determinants of elite soccer playing status among all stages of maturation, whilst vertical and horizontal-forward power performance was only established as a physiological determinant in mid- and post-peak height velocity (PHV). In addition, the isometric maximal voluntary force has shown likely and possible differences at pre- and mid-PHV in English Premier League youth soccer players (Brownlee et al., 2018). Therefore, it is worth considering the effect of biological maturation on muscular function.

Tensiomyography (TMG) has proven to be a non-invasive, valid, and reliable method for measuring skeletal muscle contractile properties through the evaluation of muscle belly radial deformation in response to an electrical stimulus (Mcgregor et al., 2018; Rey et al., 2012a; Simunic et al., 2011). In the last years, a progressive increase has been observed in studies that use TMG to evaluate contractile and mechanical characteristics in different sports populations (Garcia-García et al., 2017; Gil et al., 2015; Rey et al., 2012a). In soccer, several studies have examined the usefulness of TMG technology for analyzing the effects on muscle contractile properties in different training methods (Loturco et al., 2016; Zubac and Simunic, 2017), recovery strategies (Rey et al., 2012b), training loads throughout the competitive microcycle (Rey et al., 2020), and in establishing a neuromuscular profile (Fernández-Baeza et al., 2022; García-García et al., 2017; Rey et al., 2012a) in adult soccer players. However, little attention has been given to age’s effects on muscle contractile properties in youth soccer players. Regarding the effects of age on neuromuscular properties, Simunic et al. (2017) analyzed changes in muscle contractile properties and the fibre type in children between the ages of 9 and 14 with TMG. Those authors showed a slow- to fast-transition in the vastus lateralis (VL) when applying the relationship between myosin-heavy chain I and time contraction (Tc) in children between 6 and 10 years old, stabilizing the values of these variables until adulthood (Simunic et al., 2017). Therefore, in order to optimize the talent identification process and manage training loads in elite youth soccer players, it is essential to establish reference values of muscle contractile properties according to age.

According to our knowledge, it is still unknown how muscle contractile properties are affected by biological maturation in elite youth soccer players. Therefore, this study aimed to determine the effects of maturity status on muscle contractile properties measured by TMG and provide reference values for elite youth soccer players. Following previous investigations (Brownlee et al., 2018; Murtagh et al., 2018), it was hypothesized that there would be significant differences in TMG variables according to biological maturation stages.

## Methods

### Participants

A total of 121 elite youth soccer players (14.98 ± 1.83 years; 167.38 ± 10.37 cm; 60.65 ± 11.69 kg) with a chronological age range of 12–18 years volunteered to participate in this study. The subjects were recruited from two different academies affiliated with two professional teams which competed in Spanish first and second divisions. These players were drawn from six age categories including U13, U14, U15, U16, U17, and U18. All participants had soccer training experience from 4 to 8 years. The inclusion criteria were youth players without any injury in the previous 6 months, and who played in the outfield positions (i.e., the goalkeeper playing position was excluded from subsequent analysis). A priori power analysis was performed using G*Power software (version 3.1.9.2, Universität Kiel, Düsseldorf, Germany) (Faul et al., 2009). For power calculations (1-β), the effect size was set to 0.5 assuming a type I error of 0.05 and a type II error of 0.20 (80% statistical power) for one-way analysis of variance (ANOVA). This analysis displayed that 14 players per group would be sufficient. A parent or a legal guardian of each player signed an informed consent form allowing them to participate in this study. The benefits and risks associated with this research were explained before signing the institutionally approved informed consent form. The research protocol was approved by the Local Ethics Committee (University of Vigo; 19-0320), in accordance with the Code of Ethics of the World Medical Association (Declaration of Helsinki).

### Design and Procedures

A cross-sectional comparative study was conducted to analyze the differences in lower limb muscle contractile properties according to maturity status in youth elite soccer players. During the in-season training period, an experimental session was scheduled to carry out TMG measurements. To minimize the effects of fatigue on TMG variables, all players attended testing sessions after a recovery period of at least 72 hours. All testing sessions were conducted in the afternoon at the same time (from 17:00 to 19:00 h) and under similar environmental conditions (21–22ºC; humidity: 51–55%).

#### Anthropometric Measurements

Anthropometric measurements were carried out in accordance with the previously established procedures (Murtagh et al., 2018). The measurement of standing height was conducted with a fixed stadiometer (Holtain Limited, Crosswell, United Kingdom). In order to measure sitting height, a fixed-height table was used (Holtain Limited, Crosswell, United Kingdom). After that, the seated height was deducted from the standing height of each player to calculate lower limb length.

### Maturity Status

The biological maturation of each player was estimated using anthropometric measurements in conjunction with the chronological age through the regression equation 3 proposed by Mirwald et al. (2002) for boys. Previously, this non-invasive and time-efficient method has been used as a somatic maturity indicator among youth soccer players (Buchheit and Mendez-Villanueva, 2013; Murtagh et al., 2018). The maturity offset was defined as the amount of time (in years) before or after a participant's predicted peak height velocity (PHV). Participants were categorized into three maturity groups accordingly to the maturity offset: Pre-PHV (< −1.0 years), Mid-PHV (−0.99 to 0.99 years), and Post-PHV (> 1 years). These categories of the maturity offset were established according to the previous literature (Morris et al., 2020).

Maturity offset = −9.236 + 0.0002708

× Leg Length and Sitting Height interaction

− 0.001663

× Age and Leg Length interaction

+ 0.007216

Age and Sitting Height interaction

+ 0.02292

× Weight by Height ratio

### TMG Measurement Protocol

Rectus femoris (RF) and biceps femoris (BF) muscles were evaluated due to their crucial role during high-intensity running and technical actions in soccer (Fried and Lloyd, 1992). Youth soccer players were placed in a supine position, and a foam wedge was used to maintain a fixed knee joint angle at 120º to measure the RF. For BF measurements, a foam wedge was placed under the ankle joint in order to keep knee flexion at a 5º angle, and players were positioned in a prone position. All TMG measurements were conducted on the players' dominant leg.

Muscle belly radial displacement was analyzed by positioning a Transk-Tek digital displacement transducer (GK 40, Panpoptik d.o.o., Ljubljana, Slovenia). This displacement transducer was set perpendicular to the muscle belly, which contains a spring of 0.17 N·m^-1^. The RF and BF muscle belly were identified using the anatomical procedure developed by Perotto et al. (2005). Afterwards, the evaluator positioned two adhesive electrodes (5 x 5 cm) symmetrically to the sensor with an inter-electrode distance of 5 cm to induce an electrical stimulus. With a TMG-S1 electrical stimulator (Furlan Co. & Ltd., Ljubljana, Slovenia), a single 1-ms monophasic square wave stimulus was applied to record the change in the RF and BF muscle belly (Langen et al., 2022). For all players, the electrical stimulation began at 30 mA and was increased progressively by 10 mA. The amplitude was increased until the muscle belly reached maximal muscle belly displacement (Mcgregor et al., 2018). According to Pereira et al. (2012), a recovery period of 15 s between each pulse was established in order to avoid fatigue or post-tetanic potentiation effects. For each player, the curve with the highest muscle belly radial displacement in RF and BF muscles was considered for statistical analysis (García-García et al., 2019). All measurements were carried out by the same experienced evaluator (A.P.C.) and recorded using TMG-OK 3.0 software. The following TMG variables were used for subsequent analysis: maximal radial displacement (Dm), delay time (Td), and contraction time (Tc). In addition, the contraction velocity (Vc) was also calculated as a variable derivative from the division between Dm and the sum of Tc and Td.

### Statistical Analyses

Data are reported as means with standard deviations (SD). Effects of maturity status on TMG variables were analyzed using the statistical package R version 3.5.2 (R Core Team, 2018). The normality of data distribution was confirmed using the Shapiro-Wilk test and a histogram plot.

One-way independent-measures analysis of variance (ANOVA) and the Bonferroni post hoc test were used to explore the differences in TMG variables according to maturity status. Partial eta-squares were also calculated and the following categories were established: *η*p2 ≥ 0.01 indicated a small, ≥ 0.059 a medium, and ≥ 0.138 a large effect. Significance was established at the *p* ≤ 0.05 levels.

## Results

**Figure 1:**
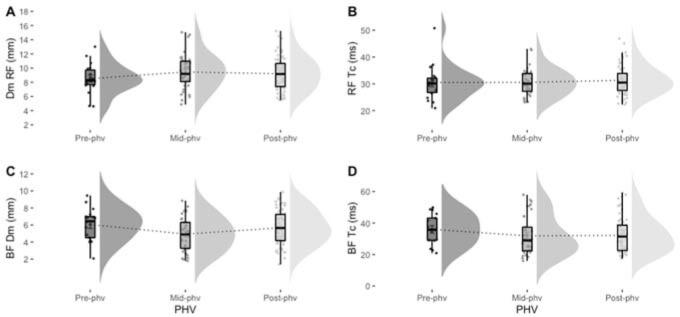
Rainclouds plot of maximal radial displacement of muscle (Dm) and time of contraction (Tc) in the rectus femoris (BF) and the biceps femoris (BF) according to maturity status in elite youth soccer players.

Figure 1 depicts descriptive values of Dm and Tc according to PHV for RF and BF muscle groups. In Table 1, one-way ANOVA showed no significant differences between PHV groups for any TMG variables in the RF: Dm (*F* = 1.005, *p* = 0.369, η_p_^2^ = 0.017), Tc (*F* = 0.327, *p* = 0.722, η_p_^2^ = 0.006), Td (*F* = 0.631, *p* = 0.534, η_p_^2^ = 0.011), and Vc (*F* = 1.159, *p* = 0.317, η_p_^2^ = 0.019). Moreover, there were no significant differences in any TMG variables in the BF: Dm (*F* = 2.463, *p* = 0.089, η_p_^2^ = 0.040), Tc (*F* = 0.911, *p* = 0.405, η_p_^2^ = 0.015), Td (*F* = 1.428, *p* = 0.244, η_p_^2^ = 0.024), and Vc (*F* = 1.877, *p* = 0.158, η_p_^2^ = 0.031).

**Table 1:** Characteristics of participants (mean ± SD) for the pre-peak height velocity (PHV), Mid-PHV and Post-PHV.

	N	Age (y)	Standing Height (cm)	Leg length (cm)	Sitting height (cm)	Body mass (kg)
Pre-PHV	18	12.57 ± 0.67	150.6 ± 6.34	72.32 ± 8.38	78.26 ± 3.69	42.93 ± 5.26
Mid-PHV	37	13.89 ± 0.87	164.0 ± 6.99	78.53 ± 5.78	85.49 ± 2.97	55.40 ± 7.54
Post-PHV	66	16.27 ± 1.26	173.1 ± 5.83	80.42 ± 5.25	93.42 ± 3.43	68.80 ± 7.21

* PHV = peak height velocity

**Table 2:** Differences in mean between TMG variables according to maturity status for rectus femoris (RF) and biceps femoris (BF) muscles.

		PHV group			ANOVA		
Muscle	Parameter	Pre-PHV (n = 18)	Mid-PHV (n = 37)	Post-PHV (n = 66)	F	*p*-Value	*ηp2*
RF	Dm (mm)	8.50 ± 2.12	9.46 ± 2.43	9.20 ± 2.31	1.005	0.369	0.017
	Tc (ms)	30.49 ± 6.65	30.53 ± 4.92	31.31 ± 5.31	0.327	0.722	0.006
	Td (ms)	24.76 ± 3.14	24.52 ± 2.34	25.09 ± 2.39	0.631	0.534	0.011
	Vc (mm/ms)	0.16 ± 0.04	0.17 ± 0.04	0.16 ± 0.04	1.159	0.317	0.019
BF	Dm (mm)	6.05 ± 1.89	4.93 ± 1.93	5.66 ± 1.98	2.463	0.089	0.040
	Tc (ms)	35.99 ± 9.62	31.85 ± 12.20	32.17 ± 10.98	0.911	0.405	0.015
	Td (ms)	23.09 ± 1.83	22.06 ± 2.36	22.40 ± 1.97	1.428	0.244	0.024
	Vc (mm/ms)	0.11 ± 0.04	0.09 ± 0.03	0.10 ± 0.04	1.877	0.158	0.031

* PHV = peak height velocity; RF = Rectus Femoris; BF = Biceps Femoris; Dm = muscle displacement; Tc = contraction time; Td = delay time; Vc = contraction velocity.

## Discussion

To the best of our knowledge, little is known about the effects of biological maturation on muscle contractile properties using TMG technology in elite youth soccer players. Therefore, the primary aim of this study was to analyze the differences in contractile and mechanical muscle properties according to biological maturation (i.e., Pre-, Mid-, and Post-PHV) in elite youth soccer players. The secondary aim of this study was to establish reference values of TMG variables in each stage of maturation. Based on previous research (Brownlee et al., 2018; Murtagh et al., 2018), it was hypothesized that biological maturation would change the muscle contractile properties. Interestingly, our data demonstrate no significant differences in Dm, Tc, Td, and Vc between different biological maturity stages in elite youth soccer players.

Specifically, TMG has been signaled as a valid alternative to more invasive analysis of skeletal muscle properties (Macgregor et al., 2018). Concretely, Tc has been determined as one of the most stable variables of TMG (Simunic et al., 2012; Tous-Fajardo et al., 2010). In addition, previous studies have established the construct validity of TMG technique to determine the proportion of the muscle fiber type (Dahmane et al., 2001; Simunic et al., 2011). In that line, Simunic et al. (2017) observed a relationship between myosin-heavy chain I and Tc. In reference to VL muscle, they found a slow-to-fast transition at the ages of 6 to 10 years. From this age for VL, the values of Tc and estimated values of myosin heavy chain I proportion stabilize showing similar values until adulthood (Simunic et al., 2017). Regarding the effects of age on the BF, the results obtained by Simunic et al. (2017) revealed similar values in Tc from the age of 12 years. In agreement with the findings of that study (Simunic et al., 2017), our results revealed no significant differences between Tc, Td, and Vc variables in the RF and BF between Pre-, Mid- and Post-PHV. Moreover, it is necessary to highlight that soccer players who composed the sample of the current study presented a chronological age from 12 to 18. At this age, Simunic et al. (2017) reported similar values of Tc in the BF and VL until adulthood.

In the soccer context, the quadriceps muscle group plays an important role in sprinting, jumping, and ball kicking, whilst the hamstrings have a key role in sprint acceleration performance and stabilize the knee during change of direction actions and tackles (Morin et al., 2015; Scurr et al., 2011). In addition, these muscle groups have been established as the most commonly injured in youth team sports athletes (Valle et al., 2018). However, despite their importance in athletic performance, it has yet been unanswered how the mechanical and contractile properties of the RF and BF may be affected by biological maturation. In this sense, a recent study conducted by Sanchez-Sanchez et al. (2020) reported non-significant differences in values of Dm, Tc and Td of the BF and RF in elite youth soccer players from three different age categories (U-14, U-16, and U-18), establishing that those contractile properties are not age-related. Similarly, our results showed that this lack of differences in contractile properties is not influenced by the maturity status either. Moreover, in both studies, youth soccer players displayed similar values of Dm in the BF and RF. Conversely, the values of Tc were lower than those obtained by Sanchez-Sanchez et al. (2020) which suggested a higher proportion of type II fibers in the RF and BF in our sample. However, youth soccer players have shown a lower Dm and higher Tc than adult professional soccer players who were shown to have higher muscle stiffness and a greater proportion of fast fibers (Gil et al., 2015; Rey et al., 2012a). Consequently, the findings of this study provide reference values of TMG variables (i.e., Dm, Tc, Td, and Vc) for BF and RF muscle groups in Pre-, Mid-, and Post-PHV elite youth soccer players.

Scientific literature has established that musculotendinous stiffness can influence muscular strength and associated characteristics such as the rate of force development and power (Secomb et al., 2015). In this respect, the Dm variable is indicative of muscle belly radial stiffness (Macgregor et al., 2018). Therefore, the Dm variations could be interpreted as a change in muscle stiffness showing modifications in muscular strength or force production. In this sense, contrary to our expectations, there were similar values of Dm for RF and BF muscle groups according to the stage of maturation in elite youth soccer players. However, previous studies have used other assessment methods to analyze maturity status effects, such as a squat jump (SJ) and a countermovement jump (CMJ) for power production (Dobbs et al., 2020; Murtagh et al., 2018), an isometric mid-thigh pull (IMTP) for maximum and rapid force production (Brownlee et al., 2018; Dobbs et al., 2020; Morris et al., 2018) or isokinetic dynamometry (Cunha et al., 2019) in different athletes’ populations.

For instance, Dobbs et al. (2020) reported that maturation produces significant improvements in absolute peak force and allometric peak force in the IMTP, SJ and CMJ in youth cricketers. These findings are also similar to those reported by Murtagh et al. (2018) in elite youth soccer players, showing that both the bilateral horizontal and vertical CMJ performances were better in Post-PHV than in Pre- and Mid-PHV. Similarly, regarding IMTP assessment, Morris et al. (2018) revealed differences in absolute PF and the rate of force development (RFD) over 100 and 300 ms between maturity offset groups, with medium to large effects (*d* = 0.56–3.8) in elite youth soccer players aged 12–18 years. When variables were allometrically scaled, the same authors observed higher values in the Post-PHV for PF and the RFD over 100 and 300 ms compared to Pre- (*d* = 0.48–0.59) and Mid-PHV (*d* = 0.24–0.44). However, only small significant differences (*d* = 0.31) were found in the RFD over 300 ms between Mid- and Pre-PHV. Likewise, in terms of isokinetic dynamometry, several studies showed that maturity status did not report a significant effect on isometric and dynamic knee extensor torque when torque values were normalised by body mass, muscle volume or the cross-sectional area (Cunha et al., 2019; De Ste Croix et al., 2002; Fukunaga et al., 2014). In consequence, practitioners should take into account that differences in muscle function according to maturity status could vary depending on the type of the method used for its assessment.

The findings of this research should be interpreted with caution due to the specific limitations derived from the data collection procedures. Even though some aspects of the current research are unique, the following limitations are to be taken into consideration. First, it is necessary to recognize that Mirwald maturation’ equation is less accurate in establishing the distance from PHV (Mirwald et al., 2002). In this sense, the use of this regression equation could underestimate the age at PHV in males under the age of 12, and overestimate the age at PHV from 16 years (Malina and Kozieł, 2014). Despite these limitations, the use of a regression equation is a practical solution in comparison with other more accurate and reliable methods such as radiography and DXA, which are more expensive and invasive (Mills et al., 2017). Second, the sample was relatively small in some biological maturity groups (i.e., Pre- and Mid-PHV). However, the sample was similar to other studies which evaluated the effects of biological maturation in youth soccer players (Cunha et al., 2019). Therefore, caution is required when interpreting these results because the lack of differences between stages of maturation could be related to the chronic adaptations due to the fact that elite youth soccer players are exposed to systematic training in a high-competitive environment and the composition of the sample (i.e., lower representation of Pre- and Mid-PHV players). Future studies should consider using a larger sample size in Pre- and Mid-PHV groups in order to provide more conclusive results. Finally, for this study elite youth soccer players were recruited, thus the results obtained cannot be inferred to other competitive levels (i.e., soccer academies or amateur clubs).

## Conclusions

In summary, this study provides normative values of mechanical and contractile muscle function for elite youth soccer players according to the biological maturation stage. According to the present findings, the assessment of neuromuscular properties in the RF and BF with TMG seems to be not compromised by maturation status of elite youth soccer players. However, the assessment of the neuromuscular profile using TMG is established as non-invasive, portable, and low-time consuming. Moreover, it does not produce fatigue, avoiding alterations in training periodization. Likewise, these data could be used to individualize and manage the training loads, reducing the risk of muscle injuries and maximizing the effect of different training regimes in elite soccer academies.

## Author Contributions

Conceptualization: E.R. and A.P.-C.; methodology: E.R., A.P.-C. and M.L.-M.; formal analysis: E.R., A.P.-C. and M.L.-M.; investigation: F.J.C.; data curation: M.L.-M. and A.P.-C.; writing—original draft preparation: A.P.-C. and M.L.-M; writing—review & editing: E.R., S.G.-V. and F.J.C. All authors have read and agreed to the published version of the manuscript.

## ORCID iD

Alexis Padrón-Cabo: 0000-0002-1077-4259

Francisco J. Corredoira: 0000-0003-3745-3030

Miguel Lorenzo-Martínez: 0000-0002-5545-8890 Sixto González-Víllora: 0000-0003-2473-5223

Ezequiel Rey: 0000-0003-4770-2694
